# Case of Symmetrical Gangrene of the Fingers

**Published:** 1878-12

**Authors:** J. T. Stewart, Noble Holton

**Affiliations:** Peoria, Ill.; Peoria, Ill.


*A Case of Symmetrical Gangrene of the Fingers.*

On the 15th of June, 1878, Mr. D. T., formerly a farmer, but not now engaged in business, aged 75 years, was attacked with bilious fever, in the early stage of which an old inguinal hernia became strangulated. The usual manipulations were made, and failed to reduce it, but in a second attempt made on the evening following were successful.

His liver was torpid, and a jaundiced condition supervened, with feeble pulse, a deranged and depressed condition of the whole system, and a disposition to diarrhœa.

He was put upon quinia, mercurials, diaphoretics, etc., until the subsidence of the fever, when he was given muriatic acid, with bitter tonics and iron, under which he improved so much that after about three weeks from the attack it was not thought necessary to continue attendance.

July 27th, called to see him again, found him sallow and depressed, his lower limbs œdematous. He said this swelling had been coming on for two weeks; pulse feeble, urine scanty, and high-colored, and diarrhœa. Upon examination of the heart, the first sound was found nearly normal, but the second was prolonged and rasping, indicating defective closure of the semilunar valves, probably due to partial ossification. He was given an infusion of digitalis, with potassium iodide and syrup of squills; also carbolic acid, tincture opium and bismuth for his diarrhœa.

The secretion of the kidneys became free and the swelling of the limbs diminished; otherwise his condition continued about the same for a week, when intense pain began in the second, third and fourth fingers of the right hand, extending up his arm to the shoulder. This pain was continuous, but much increased at night.

In a few days the tip of the third finger became livid, and soon after was gangrenous.

Carbolized oil, flaxseed meal poultices, with laudanum and various other applications, were made till it became gangrenous, when it was enveloped in brewer's yeast. The tip and the front side, as far up as the second metacarpal articulation, became sphacelated, and remained in this condition for a little time, when a line of demarcation began to form.

This line of demarcation deepened until the 24th of September the slough with a shell of the bone of the third phalanx were removed, leaving a healthy granulating surface. The reparative process went on slowly but kindly till the middle of October, when it was completely healed. A few days after the slough was removed from the finger of the right hand, the same severe pain began in the thumb of the left hand. The end soon became livid and afterwards dark purple. It was promptly enveloped in yeast well carbolized. This pain continued with greater or less severity for about a week, when it gradually subsided. The skin sloughed off, and the part healed.

October 30th, patient in fair health, is able to walk two or three miles a day, and says he enjoys life almost as well as he did in his younger days, only complaining of pain and swelling below the knees.

In Von Ziemssen's Cyclopedia, vol. vi, art. Chronic Endarteritis, page 383, we find a description of similar symptoms, due to disturbed circulation on account of disease of the arteries, and called symmetrical gangrene, because the arterial disease is bilateral.

J. T. Stewart, M. D., and

Peoria, Ill. Noble Holton, M. D.

